# Supplementary material for: Ecological relevance of flagellar motility in soil bacterial communities
Source: ISME J. 2024 Apr 22;18(1):wrae067. doi: 10.1093/ismejo/wrae067 (PMC11095265; doi:10.1093/ismejo/wrae067)
Supplement: Supplementary_Material_ISMEJ-D-24-00176_R2_submission_wrae067 [file supplementary_material_ismej-d-24-00176_r2_submission_wrae067.docx]

**Supplementary Materials**

**Ecological relevance of flagellar motility in soil bacterial communities**

Josep Ramoneda^1,2^, Kunkun Fan^3^, Jane M. Lucas^4^, Haiyan Chu^3,5^, Andrew Bissett^6^, Michael S. Strickland^7^, & Noah Fierer^1,8^

^1^Cooperative Institute for Research in Environmental Sciences, University of Colorado, Boulder, Colorado, USA; ^2^Spanish Research Council (CSIC), Center for Advanced Studies of Blanes (CEAB), Blanes, Spain; ^3^Laboratory of Soil and Sustainable Agriculture, Institute of Soil Science, Chinese Academy of Sciences, Nanjing, China; ^4^Cary Institute of Ecosystem Studies, Millbrook, New York, USA; ^5^University of Chinese Academy of Sciences, Beijing, China; ^6^CSIRO, Hobart, TAS7000, Australia; ^7^Department of Soil and Water Systems, University of Idaho, Moscow, Idaho, US; ^8^Department of Ecology and Evolutionary Biology, University of Colorado, Boulder, Colorado, USA

**Correspondence:**

Josep Ramoneda, [josep.ramoneda@ceab.csic.es](mailto:josep.ramoneda@ceab.csic.es)

Noah Fierer, [noah.fierer@colorado.edu](mailto:noah.fierer@colorado.edu)

**This PDF file includes:**

Supplementary Figures 1-9

Table S1


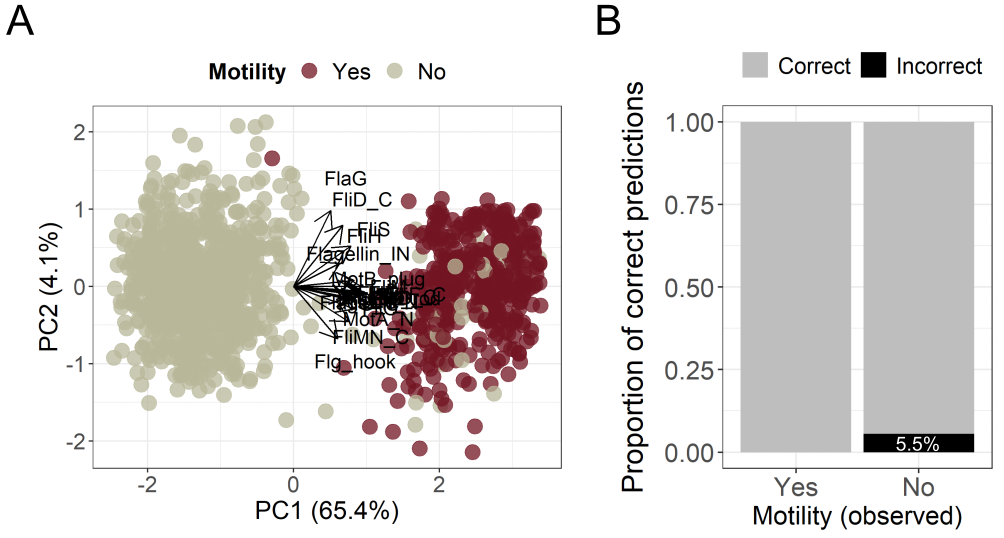
**Supplementary Figure 1. Prediction of the capacity to display flagellar motility in bacterial taxa based on presence/absence information of 21 genes involved in flagellar assembly.** A. Principal component analysis (PCA) based on the presence/absence of 21 flagellar genes in genomes of taxa that have been empirically found to be flagellated (N = 388 genomes) or non-flagellated (N = 837 genomes). Empirical information on flagellar motility was obtained from the bacterial phenotypic trait data compiled in [26]. We only included genomes that were 100% complete, contained an assembled 16S rRNA gene, and showed no signs of chimerism. B. Accuracy of a boosted regression machine learning model trained on the genomes shown in panel A for the prediction of the capacity for flagellar motility in any given bacterial genome based on the presence/absence of 21 flagellar genes. Accuracy was tested on 30% of the original genome set (116 genomes from flagellated taxa and 251 genomes from non-flagellated taxa). Genomes were obtained from the Genome Taxonomy Database (GTDB r207; [27]).

**Supplementary Figure 2. Taxonomic distribution of genomes with empirically determined capacity for flagellar motility that were used as training data for a boosted regression machine learning model to predict the capacity for flagellar motility based on the presence/absence of 21 flagellar genes.** Flagellar motility information was obtained from the bacterial phenotypic trait data compiled in [26]. N_Training set_ = 858 genomes, N_Full set_ = 1225.


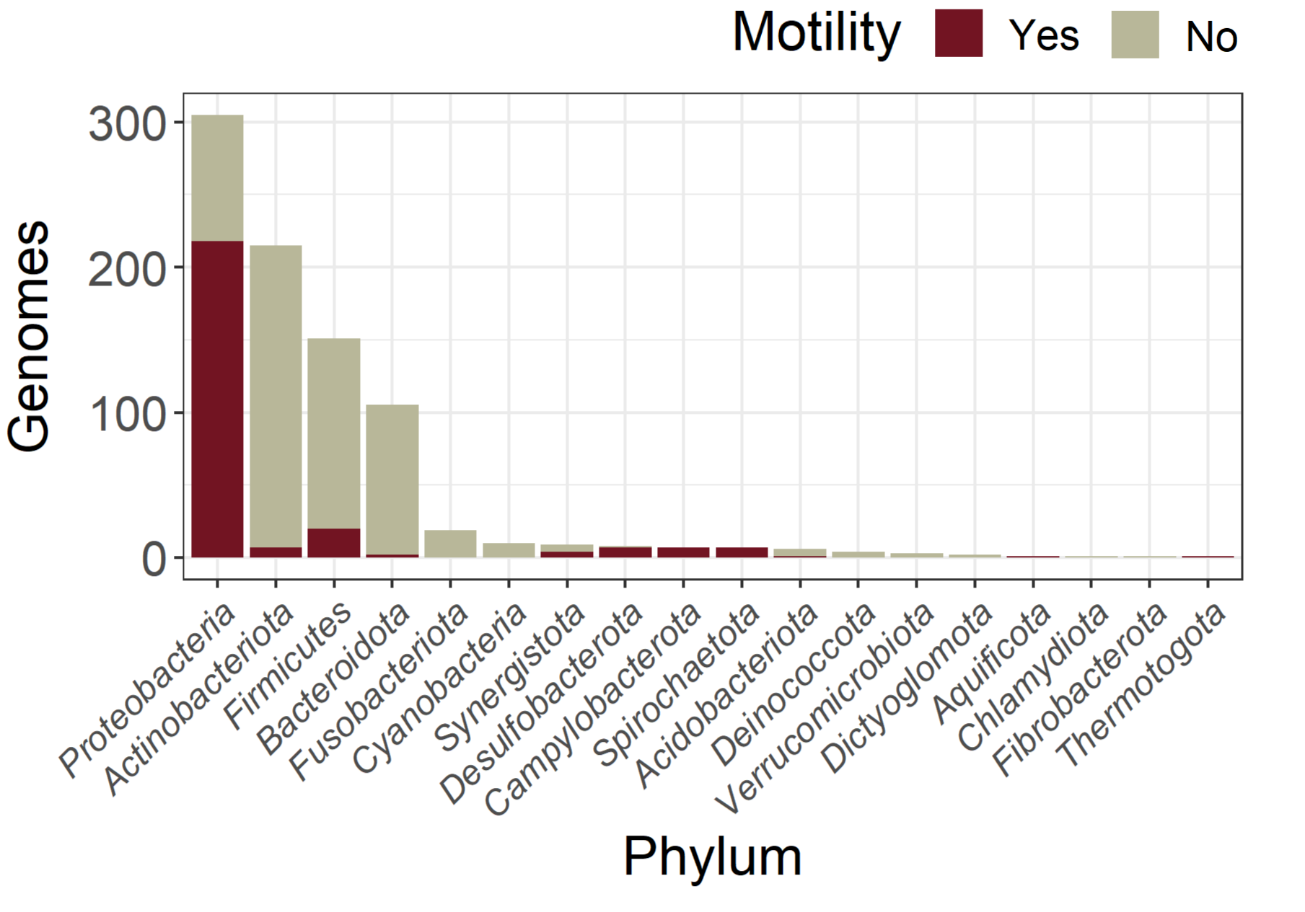


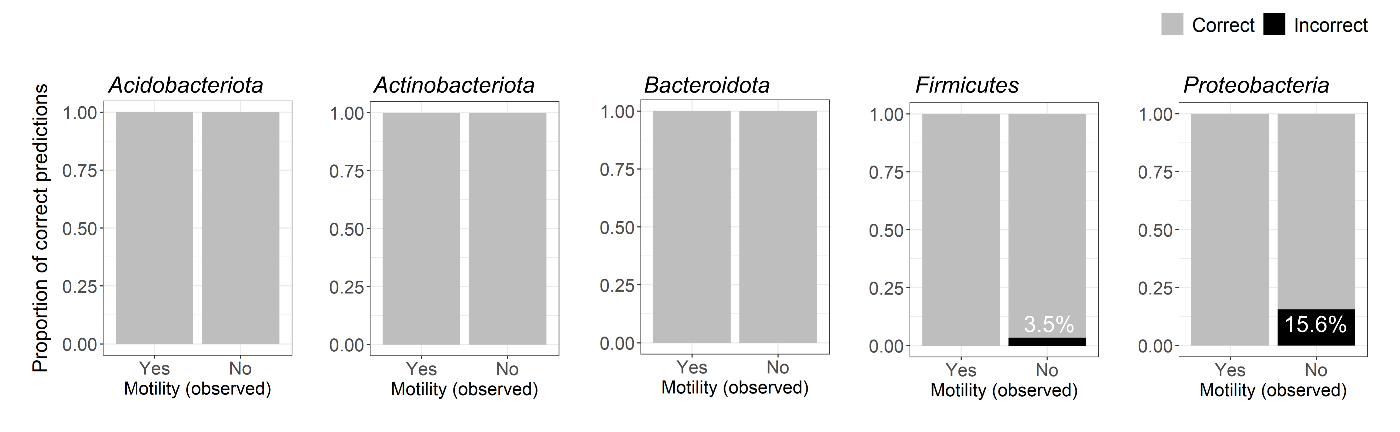
**Supplementary Figure 3. Predictive accuracy across phyla of a boosted regression machine learning model for the prediction of the capacity for flagellar motility in any given bacterial genome based on the presence/absence of 21 flagellar genes.** Accuracy was tested on 30% of the original genome set (116 genomes from flagellated taxa and 251 genomes from non-flagellated taxa). Genomes were obtained from the Genome Taxonomy Database (GTDB r207; [27]). N*_Acidobacteriota_* = 8, N*_Actinobacteriota_* = 87, N*_Bacteroidota_* = 52, N*_Firmicutes_* = 66, N*_Proteobacteria_* = 123.

**
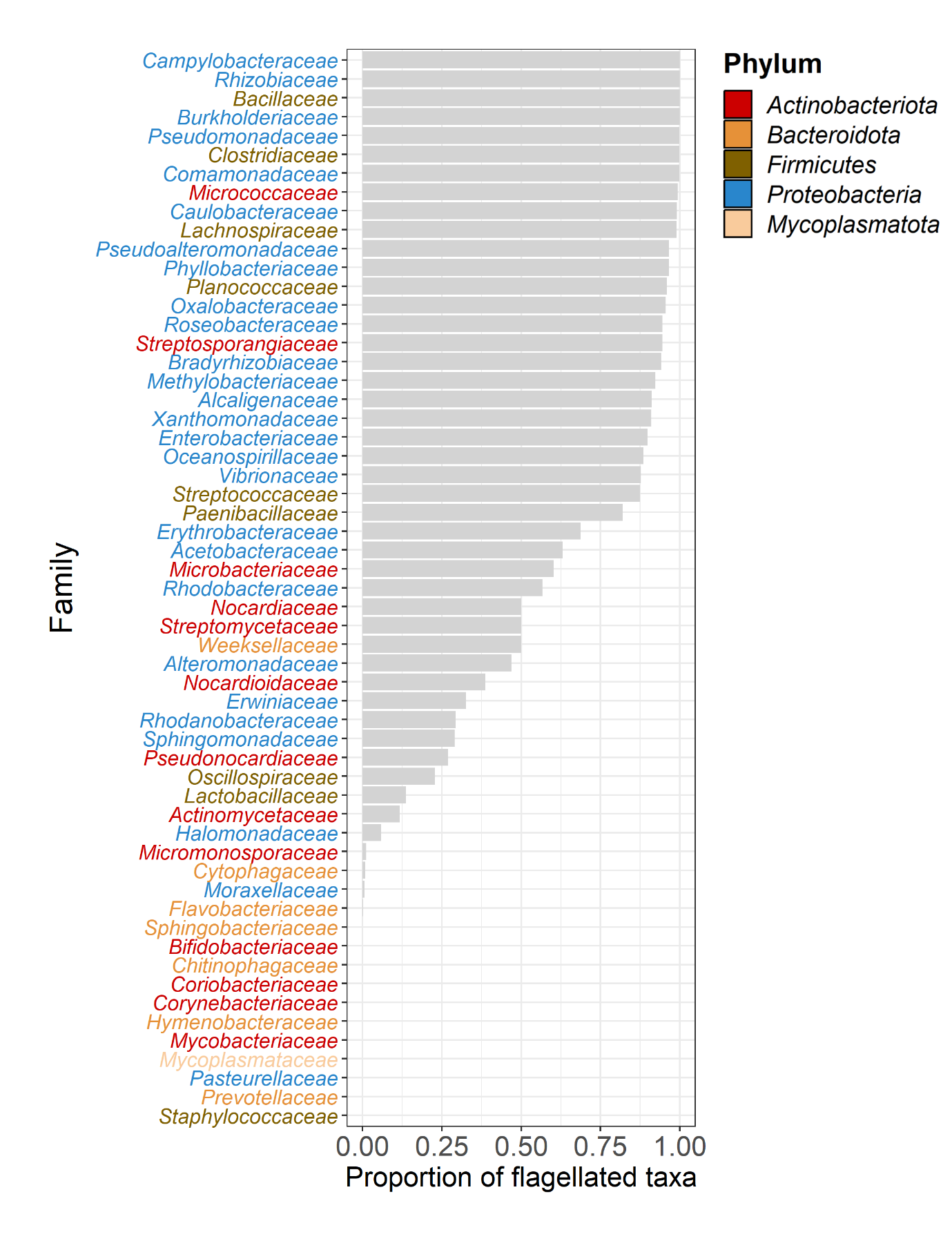
Supplementary Figure 4. Prevalence of flagellar motility across bacterial families containing more than 100 high-quality genomes in the Genome Taxonomy Database (GTDB r207; [27]).** We only included genomes that were >95% complete, contained an assembled 16S rRNA gene, and showed no signs of chimerism. N = 23,256 genomes.

**Supplementary Figure 5. Variation in flagellar motility status across taxonomic ranks.** A measure of variation in the flagellar motility status of taxa belonging to different taxonomic ranks was obtained from the standard deviation (SD) of their flagellar motility status (1, flagellated; 0, non-flagellated). Numbers in brackets indicate the total number of unique taxa within each of the taxonomic ranks. Red diamonds indicate the mean of the standard deviation of the flagellar motility status within each taxonomic rank.

**
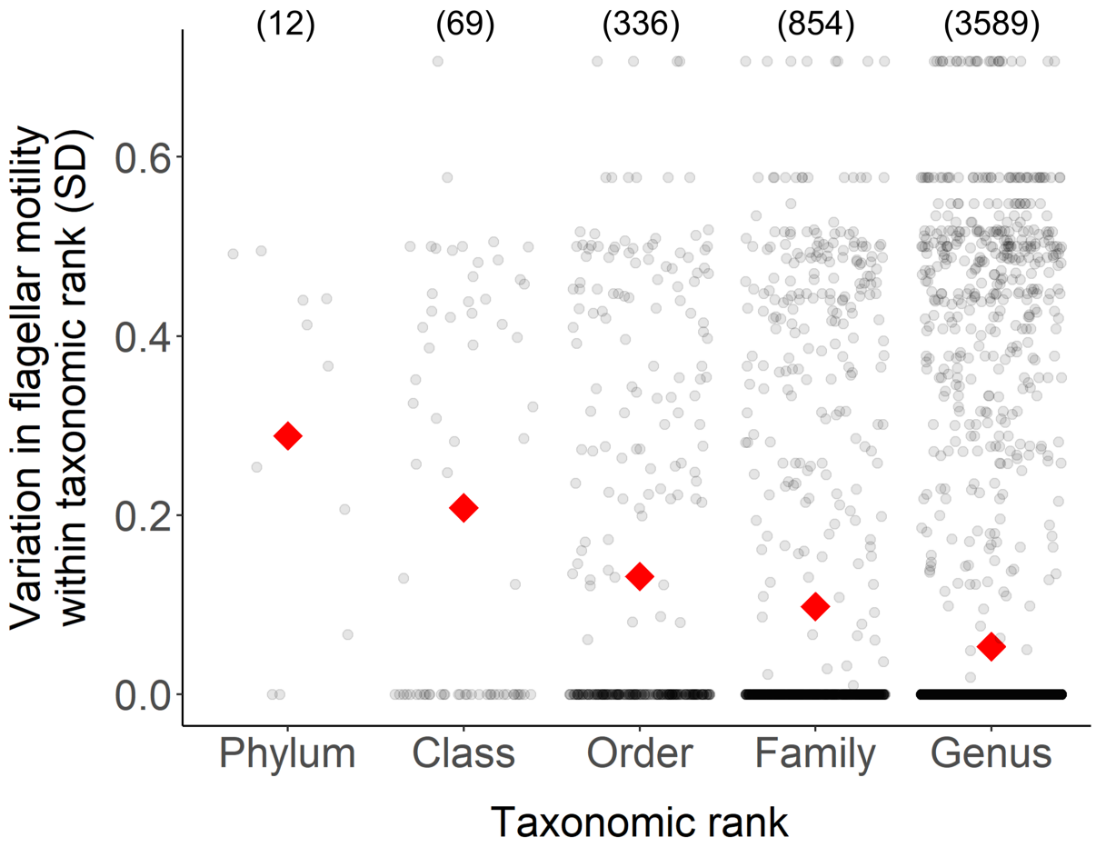
**

**Supplementary Figure 6. Distribution of the total number of 16S rRNA gene copies per genome and genome size across the 6 phyla with even proportions of taxa predicted to be flagellated and non-flagellated.** A. Number of 16S rRNA gene copies in genomes of taxa predicted to be flagellated and non-flagellated. B. Genome size of taxa predicted to be flagellated and non-flagellated. Statistical significance was obtained from Mann-Whitney U tests (*P* < 0.05), N = 21,551.

**
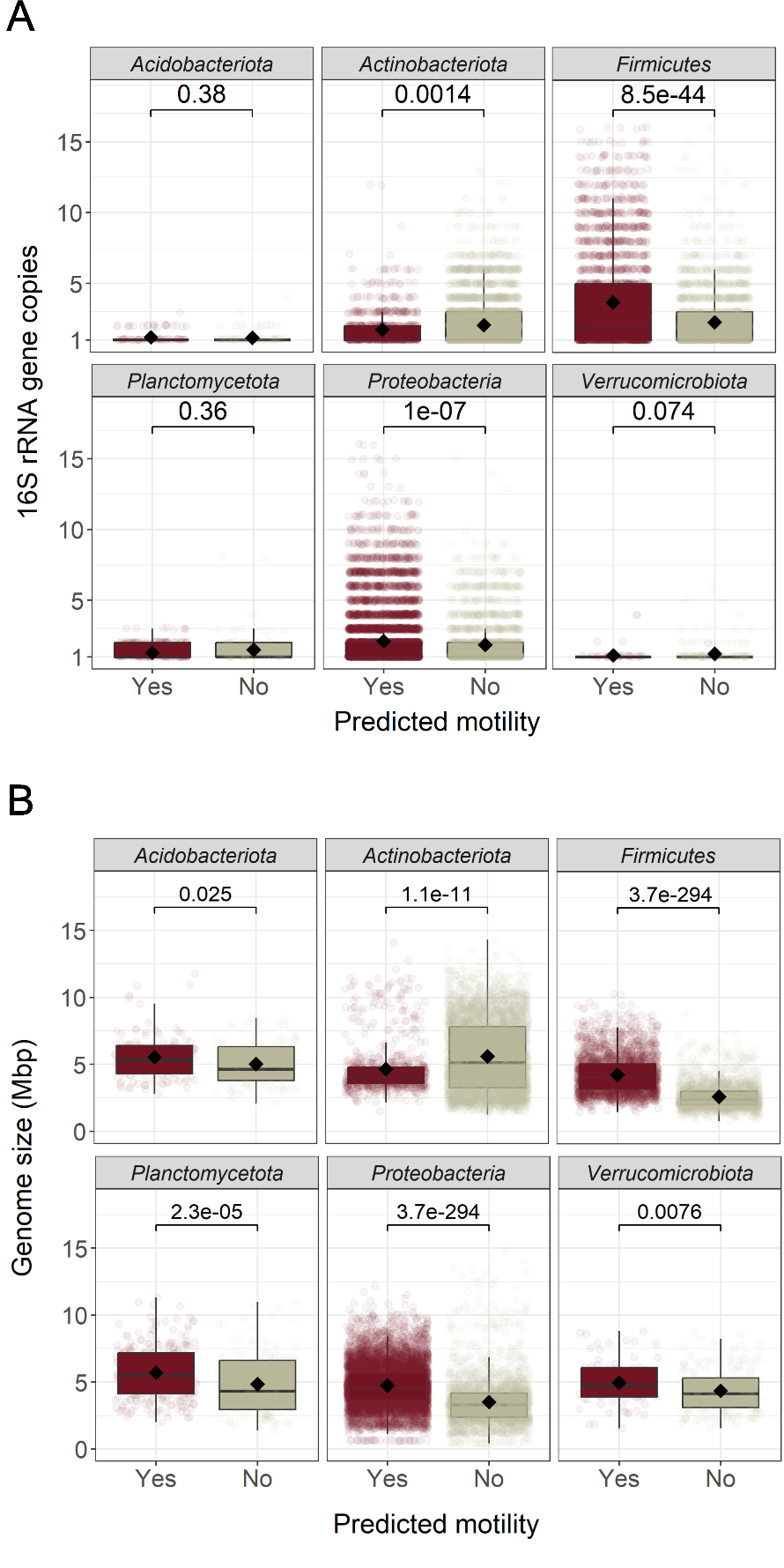
**

**Supplementary Figure 7. Estimated prevalence of flagellar motility in bacterial communities from 9 soil depth profiles collected across the USA (Surface, 0-20cm; Subsurface, 20-90cm, N = 66; [64]).**

**
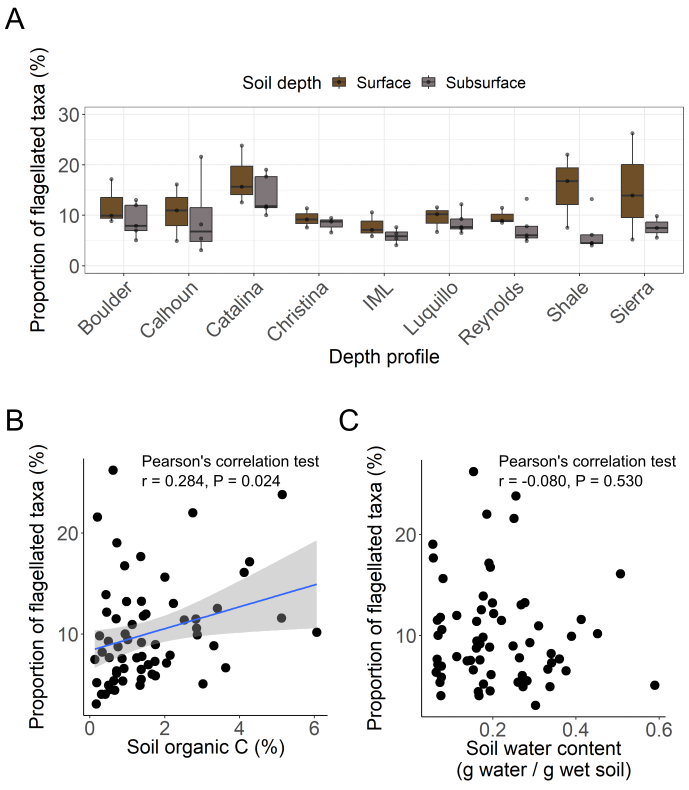
**

**Supplementary Figure 8. Difference in the prevalence of flagellar motility in rhizosphere and bulk soil bacterial communities collected from citrus species across the globe (N = 10; [67]).**

**
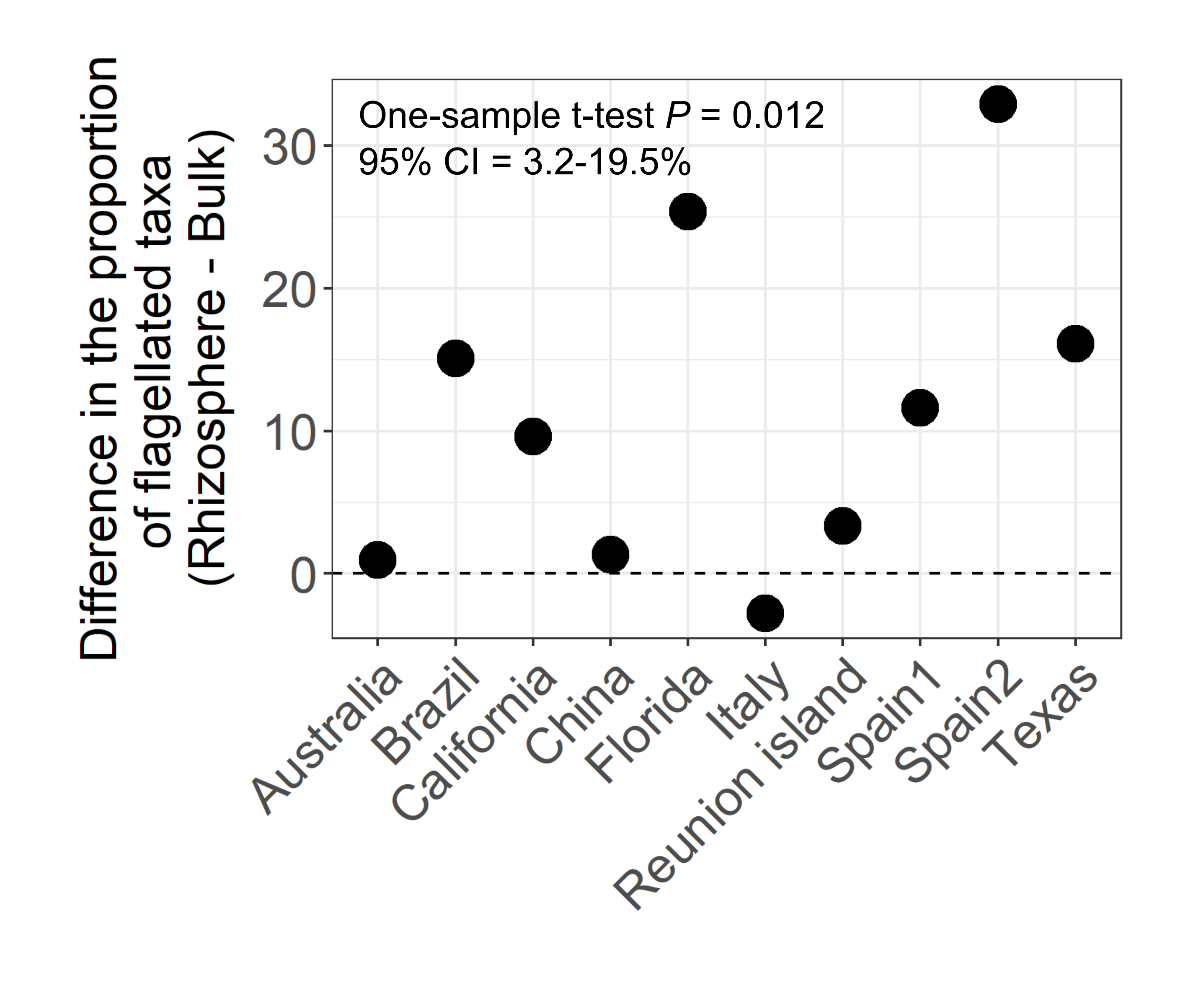
**

**Supplementary Figure 9. Taxonomic composition of the Amplicon Sequence Variants (ASVs) that responded to glucose amendment in soil.** Bacterial communities from a 117 day soil incubation experiment with daily glucose amendment were characterized using amplicon sequencing of the 16S rRNA gene [70]. ASVs were considered responsive to glucose amendment based on a differential abundance analysis comparing bacterial communities from soils amended with glucose versus communities from soils that did not receive any external carbon inputs (N = 28 responsive ASVs).

**
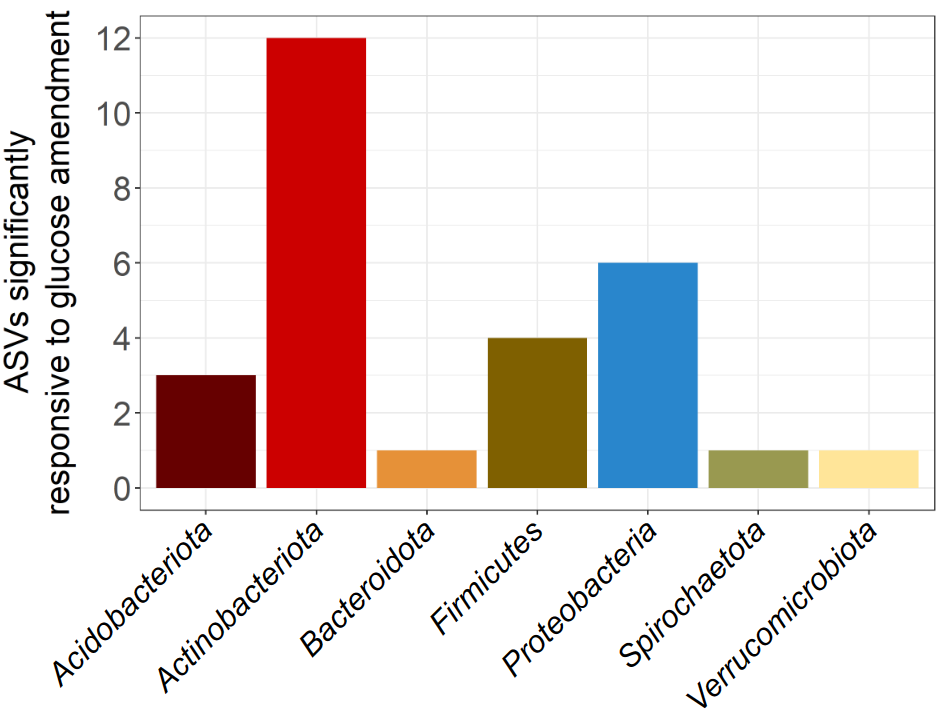
**

**Table S1. Summary of the shotgun sequencing reads generated from a soil incubation experiment where soils were amended or not with glucose over a 117-day period [70].**

| **Sample ID** | **SRA accession** | **Glucose addition** | **Sequenced reads** | **Quality filtered reads** |
| --- | --- | --- | --- | --- |
| Jar_1 | [SRR27830851](https://trace.ncbi.nlm.nih.gov/Traces?run=SRR27830851) | No | 69987711 | 64408229 |
| Jar_2 | [SRR27830850](https://trace.ncbi.nlm.nih.gov/Traces?run=SRR27830850) | No | 78670031 | 72015854 |
| Jar_3 | [SRR27830849](https://trace.ncbi.nlm.nih.gov/Traces?run=SRR27830849) | No | 71350005 | 65144468 |
| Jar_4 | [SRR27830848](https://trace.ncbi.nlm.nih.gov/Traces?run=SRR27830848) | No | 73697307 | 67586296 |
| Jar_5 | [SRR27830847](https://trace.ncbi.nlm.nih.gov/Traces?run=SRR27830847) | No | 91881320 | 84293465 |
| Jar_26 | [SRR27830846](https://trace.ncbi.nlm.nih.gov/Traces?run=SRR27830846) | Yes | 88518089 | 81320265 |
| Jar_28 | [SRR27830845](https://trace.ncbi.nlm.nih.gov/Traces?run=SRR27830845) | Yes | 81752275 | 74119288 |
| Jar_29 | [SRR27830844](https://trace.ncbi.nlm.nih.gov/Traces?run=SRR27830844) | Yes | 65392095 | 60209247 |
| Jar_30 | [SRR27830843](https://trace.ncbi.nlm.nih.gov/Traces?run=SRR27830843) | Yes | 143506441 | 131794731 |
